# *Vitex Agnus-Castus* Extract Supplementation Enhanced Growth Performance, Hemato‐Biochemical Parameters, Intestinal Histomorphometry, Flesh Composition, and Quality of Nile Tilapia, *Oreochromis niloticus*

**DOI:** 10.1155/anu/2600670

**Published:** 2026-01-22

**Authors:** Ahmed Ismail Mehrim, Mohamed Moaaz Refaey, Abdallah Tageldein Mansour, Ehab El-Haroun, Osama Awad Zenhom, Hamada Antar Areda

**Affiliations:** ^1^ Animal Production Department, Faculty of Agriculture, Mansoura University, 35516, Al-Mansoura, Egypt, mans.edu.eg; ^2^ Animal and Fish Production Department, College of Agricultural and Food Sciences, King Faisal University, Al-Ahsa, Saudi Arabia, kfu.edu.sa; ^3^ Fish and Animal Production Department, Faculty of Agriculture (Saba Basha), Alexandria University, Alexandria, 21531, Egypt, alexu.edu.eg; ^4^ Department of Integrative Agriculture, College of Agriculture and Veterinary Medicine, United Arab Emirates University, P.O. Box 15551 Al Ain, Abu Dhabi, UAE, uaeu.ac.ae; ^5^ Central Laboratory for Aquaculture Research, Agriculture Research Center, Abbasa, Abou Hammad, Egypt, arc.sci.eg; ^6^ Department of Animal, Poultry and Fish Production, Faculty of Agriculture, Damietta University, Damietta, 34517, Egypt, du.edu.eg

**Keywords:** chaste tree extract, flesh quality, growth performance, intestinal histomorphometry, Nile tilapia, physiological responses

## Abstract

Plant‐derived extracts offer a promising alternative to synthetic additives in fish feed, with the aim of enhancing growth, health, and sustainable production. The present study aimed to evaluate the effect of the hydroalcoholic extract of dietary Chaste tree, *Vitex agnus-castus* (VAC), on growth performance, feed utilization, digestive enzymes, hematological, serum biochemical parameters, intestinal histomorphometry, and flesh composition and quality of Nile tilapia, *Oreochromis niloticus*. A total of 160 Nile tilapia fingerlings were allocated in five equal treatments and fed increasing levels of VAC extract (0, 5, 10, 15, and 20 g/kg of diet) and referred as control, VAC_5_, VAC_10_, VAC_15_, and VAC_20_, respectively, for 8 weeks. Fish in the VAC_5_ group achieved the highest growth performance, digestive enzyme activities, and significantly improved feed conversion ratio (FCR) and protein efficiency ratio (PER). The predicted maximum VAC level supplementation was 9.81 g kg^–1^ diet as determined by quadratic regression based on growth and feed utilization. The hematological parameters increased significantly in the VAC_5 and 10_ groups. Liver function enzymes, glucose (GLU), triglycerides (TGs), and creatinine were significantly decreased, while total protein (TP) and albumin (ALB) were significantly increased in fish fed VAC_5_ and VAC_10_ diets. However, total cholesterol (TCH) was significantly increased with VAC supplementation on a level‐dependent basis. Histometric investigation of the intestinal tract revealed a significant improvement in muscular and submucosal thickness, villi length, and width. The chemical composition of the muscle revealed an improvement in protein and dry matter in the VAC_5_ group, with decreased water loss during storage, dripping, and freezing. In conclusion, VAC extract can be used as a feed additive in the Nile tilapia diet at levels of 5–10 g kg^–1^ diet with a positive response in growth, physiological response, and histological architecture.

## 1. Introduction

Around the world, aquaculture is becoming a more significant and integral part of ecological and agricultural ecosystems [1]. By 2022, aquaculture accounted for 59% of global fisheries and aquaculture production, with 130.9 million tons valued at USD 312.8 billion, representing a continuous increase in the sector’s share of global fish output [2]. In addition, aquaculture has a clear obligation to increase the socio‐economic impact of some areas because it not only provides critical nutrients but also creates a variety of job opportunities [3]. Particularly, freshwater aquaculture is one of the major and fastest‐growing food‐producing sectors in the world. It is a significant substitute for expensive animal protein in developing nations [4]. To ensure the long‐term sustainability of aquaculture, it is necessary to enhance fish growth, quality, and maintain less environmental impact [5, 6]. Tilapia, a cichlid fish species, is a main economic freshwater fish extensively farmed worldwide due to its ability for aquaculture, high palatability, and rapid growth rate [2]. Eleven species of tilapia are cultivated in Africa [7]; however, Nile tilapia, *Oreochromis niloticus*, is considered one of the most productive farmed fish in Egypt and worldwide [8].

To enhance growth performance and manage microbial infection, antibiotics were often utilized as feed additives in commercial aquaculture [9]. Nevertheless, one of the most significant negative effects of chemotherapy is the increase of antibiotic‐resistant bacteria, as well as the likelihood of modifying resistant genes of human pathogens [10]. Therefore, several efficient alternative approaches have been proposed, including immunostimulants, vaccination, probiotics, medicinal plants, and other natural feed supplements [11, 12]. Medicinal plants and their extracts are one of ecofriendly ways for enhancing animal performance, whereas it is accessible locally, affordable, effective against a variety of diseases, and biodegradable [13, 14].

Additionally, medicinal plants have a wide range of bioactive components, including phenols, alkaloids, flavonoids, tannins, terpenoids, saponins, glycosides, and essential oils, which have been shown to enhance immune responses, increase disease resistance, and stimulate fish performance [15]. The intervention of medicinal plants or their extracts in aquafeed exhibits several positive characteristics as immunostimulants, growth promoters, appetite stimulants, antioxidants, antibacterial, and anti‐inflammatory compounds [13]. It has been demonstrated in several studies that various extracts from medicinal plants can improve immunological–physiological responses and growth performance [16–19], increase antioxidant defense [20, 21], protect against stress or toxicity [22, 23], and enhance disease resistance [24, 25] not only for tilapia (*Oreochromis* sp.) but also to improve the health and productivity of other fish species [26, 27].

*Vitex agnus-castus* (VAC; family *Lamiaceae*) is a typical medicinal plant that grows all over the world, from Central Asia and Southern Europe to the Mediterranean area [28]. Historically, VAC has been used to treat inflammatory bowel disease, menopause, hyperprolactinemia, corpus luteum insufficiency, premenstrual syndrome, menstrual problems, and disturbed breastfeeding [29]. It has immunostimulant, antimicrobial, and antioxidant characteristics [30, 31]. In addition, several studies have demonstrated that the dietary addition of VAC increased growth performance of Zebrafish, *Danio rerio* [32, 33], goldfish, *Carassius auratus* [34], immune responses of *C. auratus* [34, 35], resistance to different pathogenic bacteria of *C. auratus* [35], and common carp, *Cyprinus carpio* [36]. The medicinal portions of VAC (fruits and leaves) also contain antifungal [37], antioxidant [30], antimicrobial [38], and antimutagenic properties [39]. Flavonoids, essential fatty acids, triterpenes, iridoid glycosides, diterpenoids, tannins, alkaloids, and essential oils are among the significant phytochemical substances that provide VAC biological advantages [31]. Recently, the effect of VAC was determined on tilapia using commercial synthetic extract with low inclusion levels up to 1%. The results indicate appositive effects of using VAC on growth, hematological, and biochemical properties of Nile tilapia [40]. According to the best of our knowledge, there are limited comprehensive studies evaluating the optimum supplementation levels of VAC extract in Nile tilapia, *O. niloticus* diet. Therefore, the current study aims to determine the optimum dietary level of VAC hydroalcoholic extract for *O. niloticus* fingerlings on growth performance, feed efficiency, digestive enzymes, hemato‐biochemical markers, and intestine histomorphometry, as well as flesh composition and quality properties.

## 2. Materials and Methods

### 2.1. Preparation of the Herbal Extracts

VAC was obtained from a local store for medicinal and aromatic plants in Mansoura city, Dakahlia Governorate, Egypt. Using the soaking method, 50 g of dried fruits and VAC leaves was ground into a powder and then added to 1500 mL of solvent (a mixture of equal parts of distilled water and ethanol). The mixture was stirred at 90 rpm for 48 h until it became uniform. The solution was filtered through double layers of medical gauze (~20–100 µm), and then the solvent was evaporated using a rotary evaporator (Heidolph WD 2000, Schwabach, Germany). Finally, the pure hydroalcoholic extracts of VAC were stored in a refrigerator at 4°C.

### 2.2. Gas Chromatography–Mass Spectrometry (GC–MS/MS) Analysis of VAC

GC–MS analysis of samples was performed using TRACE 1310 GC ‐ TSQ 9000 Triple Quadrupole MS (Thermo Scientific, Austin, TX, USA) with a direct capillary column triglycerides (TGs) ‐ 5 MS 30 m × 0.25 mm × 0.25 µm film thickness. Helium gas was used as carrier gas and was adjusted to column velocity flow of 1.0 mL/min. Spectra were collected, and the components were identified by comparison of their retention times and mass spectra with those of WILEY 09 and NIST 14 mass spectral database [41]. The active components of VAC are presented in Table 1.

**Table 1:** Active components of VAC as revealed by GC–MS.

Compound name	Molecular formula	Chemical class	Functional effect	Molecular weight	Area(%)	Retention time (min)
Undecane	C_11_H_24_	Alkane hydrocarbon	Anti‐inflammatory	156	1.36	12.50
Benzaldehyde,2,4‐dimethyl‐	C_9_H_10_O	A colorless aromatic	Antioxidant	134	1.68	16.09
Benzaldehyde, 4‐methoxy‐	C_8_H_8_O_2_	Aromatic compound	Antimicrobial	136	1.47	17.24
Iberin nitrile	C_5_H_9_NOS	A natural compound	Antimicrobial	131	12.89	20.51
*n*‐Hexadecanoic acid (palmitic acid)	C_16_H_32_O_2_	Straight‐chain saturated fatty acid	Energy, metabolism, and immunity	256	32.45	33.79
Linoleic acid ethyl ester	C_20_H_36_O_2_	A plant‐derived fatty acid ester	Anti‐inflammatory	308	1.65	36.96
Ethanol,2‐(9,12‐octadecadienyloxy)‐, (*Z,Z*)‐	C_20_H_38_O_2_	A fatty alcohol ether	Antioxidant, antimicrobial, and anticancer agent	310	1.65	36.96
8,11,14‐Eicosatrienoic acid, (*Z,Z,Z*)‐	C_20_H_34_O_2_	A polyunsaturated fatty acid	Anti‐inflammatory, blood clotting, and cell signaling	306	1.65	36.96
Oleic acid	C_18_H_34_O_2_	A monounsaturated omega‐9 fat	Anti‐inflammation and lowering bad cholesterol	282	21.22	37.08
Octadecanoic acid	C_18_H_36_O_2_	A versatile fatty acid	Metabolic processes, providing energy and structural support	284	8.36	37.47
Ethyl iso‐allocholate	C_26_H_44_O_5_	A steroid‐like compound	Antimicrobial and anticancer	436	1.20	42.47
9,12,15‐Octadecatrienoic acid, 2‐([trimethylsilyl]oxy)‐1‐([(trimethylsilyl) oxy]methyl) ethyl ester, (Z,Z,Z)‐	C_27_H_52_O_4_Si_2_	A linolenic acid monoester	Antioxidant and anti‐inflammatory	496	1.20	42.47
Hexadecanoic acid, 2‐hydroxy‐1‐(hydroxyl methyl) ethyl ester	C_19_H_38_O_4_	Fatty alcohol ether	Antioxidant and anti‐inflammatory	330	5.26	43.21
Hexadecanoic acid, 1‐(hydroxymethyl)‐1,2‐eth anediyl ester	C_35_H_68_O_5_	Fatty alcohol ether	Anti‐inflammatory, antioxidant, and hepatoprotection	568	5.26	43.21
Hexadecanoic acid, 2,3‐dihydroxypropyl ester	C_19_H_38_O_4_	Fatty alcohol ether	Antimicrobial, antioxidant, or anticancer	330	5.26	43.21
Octadecanoic acid, 2‐hydroxy‐1‐(hydroxyl methyl) ethyl ester	C_21_H_42_O_4_	Fatty alcohol ether	Anti‐inflammatory	358	9.51	47.16

### 2.3. Fish Management

The *O. niloticus* fingerlings were maintained in a plastic tank for 2 weeks period as adaptation. During this time, fish were fed a control diet (commercial diet; 32% protein, 6.20% fat, and 5.70% fiber), which was purchased from New Hope Egypt Aquatic Technology Co., Ltd, Egypt. Afterwards, 160 *O. niloticus* fingerlings with an average initial body weight of 8.0 *g* ± 0.02 were randomly assigned to five treatments in 20 glass aquariums (length 80 cm × width 35 cm × height 40 cm). Each treatment was applied in four aquariums, with eight fish per aquarium. One‐third of the water in each aquarium was replaced daily with fresh water after removing fish feces and waste by siphoning. Water quality was monitored weekly and maintained at optimum levels for tilapia as follows: DO (5.20 ± 0.65 mg/L), temperature (26.40 ± 2.22°C), and pH 7.92 ± 0.54. Feed was manually introduced to fish twice daily at 9:00 a.m. and 3:00 p.m for 60 days at a level of 5% of live body weight. For better feed intake determination, the remaining pellets were collected after 30 min of diet introduction, dried, weighed, and subtracted from the offered ration. Every 15 days, all fish in each aquarium were weighed to adjust the daily feed amount. The handling and experimental procedures involving all animals were carried out following the “Ethics and Guidelines for the Use of Experimental Animals in Scientific Research,” Mansoura University, Egypt (MU ‐ ICUC).

### 2.4. Experimental Treatments and Diets

Fish in the current study were divided into five experimental groups. The first group received a control diet with a free VAC extract, while the other four treatments were fed diets supplemented with 5, 10, 15, and 20 g of VAC extract kg^−1^, labeled as (VAC_5_), (VAC_10_), (VAC_15_), and (VAC_20_), respectively. The VAC extract was added to diets at the respective doses after crushing and mixed well until homogenous. The mixture was moistened using distilled water at room temperature (25°C) and re‐pelleted again using meat mincer (2 mL size), dried in forced air in an oven until gaining constant weight. The pellets were stored at 5°C until use. The proximate chemical composition of the used commercial diet was not affected by repleting and remaining isonitrogenous/isoenergetic with the proximate chemical composition of 32% protein, 6.20% fat, and 5.70% fiber.

### 2.5. Tested Parameters

#### 2.5.1. Growth Performance and Feed Efficiency Parameters

The growth performance and feed utilization criteria were calculated according to the following equations:

– Weight gain (WG; g fish^–1^) = final weight (FW) (g) – initial weight (IW) (g)
– Average daily gain (ADG; g fish^–1^ day^–1^) = WG (g) / T (days).
– Body weight index (BWI; %) = ([(FW – IW) / IW] × _10_0)
– Specific growth rate (SGR; % day^–1^) = _10_0 (ln FW – ln IW/T).

where ln: natural log; IW: initial weight (g); FW: final weight (g); T: the experimental period (days).

– Feed conversion ratio (FCR) = total feed intake (g) / WG (g).
– Protein efficiency ratio (PER) = body WG (g) / protein intake (g).

#### 2.5.2. The Blood Sampling and Hemato‐Biochemical Parameters

Eight fish (24 h fasted) from each treatment were randomly selected at the end of the experimental period and treated with clove oil extract for anesthesia (50 mL of commercial alcoholic clove oil extract in 10 L of water). Subsequently, blood was extracted from the caudal peduncles utilizing disposable 5 mL syringes. The obtained blood samples were split into two portions: the first portion was placed in heparinized plastic tubes for hematological analysis. According to Decie and Lewis [42], the hematological variables, including hemoglobin (Hb), total red blood cells (RBCs), packed cell volume (PCV), blood platelets (PLTs), and total white blood cells (WBCs), were assessed using the collected whole blood samples. In accordance with [43], blood indices, comprising mean cell volume (MCV), mean cell hemoglobin (MCH), and MCH concentration (MCHC) were calculated. Blood performance (BP) = Ln Hb (g/dL) + Ln Ht (%) + Ln RBC (∗10^5^/mm^3^) + Ln WBC (∗10^3^/mm^3^) + Ln total protein (TP) (g/L) [44].

Blood samples were also collected in dry plastic tubes and then subjected to centrifugation for 15 min at a speed of 3500 rpm to separate the blood serum. Until the biochemical assays were completed, the serum samples were kept at –20°C in a deep freezer. Liver function parameters, including aspartate aminotransferase (AST) and alanine aminotransferase (ALT) activities, were determined through the use of commercial test kits (Humalyzer 3000, Human, Germany). TP and albumin (ALB) were also measured by commercial kits according to Tietz [45] and Doumas et al. [46], respectively, while globulin (GLB) was calculated by subtracting ALB from the TP. Serum kidney function parameters such as creatinine and uric acid (UA) were determined according to Tietz [45]. Serum lipid profiles such as total cholesterol (TCH) and TGs were measured by McGowan et al. [47]. Serum glucose (GLU; as a stress biomarker) was measured according to Henry [48].

#### 2.5.3. Digestive Enzyme Activities

The activity of amylase was quantified as the release of 1 μmol of maltose per minute at a wavelength of 540 nm, equivalent to one unit of activity as established by Bernfeld [49]. The activity of lipase was assessed through the titration method employing an emulsion of olive oil and gum Arabic, as described by Worthington [50]. The activity level was characterized by the release of 1 μmol of fatty acid per minute, constituting one unit of activity.

#### 2.5.4. Histomorphometry Examination of the Intestine

For the purpose of histological examination, tissue samples (*n* = 6 per treatment) from the mid‐intestinal region were procured and subsequently preserved in a 10% neutralized formalin solution. The samples underwent a dehydration process utilizing a series of graded alcohol concentrations (70%, 85%, 96%, and 99%). Following this, the samples were cleared with xylene prior to being embedded in paraffin wax. As stated by Bancroft and Gamble [51], wax blocks were sectioned to a thickness of six microns and subsequently stained with hematoxylin and eosin (H&E). The histometric parameters were assessed in accordance with the methodology outlined by Radu‐Rusu et al. [52].

#### 2.5.5. Muscular Chemical Composition and Flesh Quality Parameters

Fish dorsal muscles (*n* = 6 per treatment) were obtained after blood samples were collected in order to ascertain the parameters related to meat quality and chemical makeup. According to AOAC [53], muscle samples were collected for proximate chemical composition and stored at –20°C until the chemical analysis was completed. The metrics used to gauge flesh quality metrics included water holding capacity (WHC), stored loss (SL), drip loss (DL), and frozen leakage rate (FLR). WHC was determined by weighing a dorsal muscle sample, placing it between two filters, and applying 3.5 kg weight for 15 min. The difference between the two weights as a percentage of the fresh weight is used to estimate the WHC. Five fish fillets (average weight: 5.0 ± 0.5 g) were used for each treatment in order to calculate the DL of fish muscle. In accordance with AOAC [53], fillets were put in plastic bags and kept at 4°C for 72 h in order to measure DL by the following equation:

DL%=W01−W/W0×100

where: W0 is the weight of the fillet sample before storage; W1 is the weight after storage (3 days).

In accordance with LingQiao et al. [54], the SL and FLR were calculated. In order to calculate SL and FLR, 10 fillet samples for each treatment in each interval period were weighed at 5.0 ± 0.5 g, packaged in plastic bags, and then separated into two sets. The samples were then kept at 4°C for 24 h and –20°C for 0, 1, and 2 h, respectively. According to the proportion of IW loss, SL and FLR were computed.

### 2.6. Statistical Analysis

The data were presented as mean ± SE. Data normality was tested using Kolmogorov–Smirnov [55]. A one‐way ANOVA was performed by SAS software version 9.1.3 for Windows [56] to assess treatment effects. The quadratic regression was conducted among dietary VAC levels and FW and SGR. Ratios and percentages were arcsine‐transformed for analysis. Tukey’s post hoc test compared means, with significance at *p* ≤ 0.05.

## 3. Results

### 3.1. Growth and Feed Utilization

Compared to the control group, the VAC supplementation had substantially increased *O. niloticus* FW, WG, ADG, BWI, and SGR (*p* < 0.05). Fish in the VAC_5_ group achieved the highest growth performance parameters among all groups (Table 2). Regarding the feed utilization parameters, *O. niloticus* in the VAC_5_ significantly experienced improved FCR and PER among all groups (*p* ≤ 0.05). FI did not significantly differ among all groups (*p* ≥ 0.05; Table 2). From the quadratic analysis shown in Figure 1, the predicted maximum VAC extract level for *O. niloticus* FW and SGR is 9.81 g kg^–1^ diet as a statistical trend under experimental conditions, not an absolute biological optimum.

Figure 1Quadratic regression model of (A) final weight and (B) specific growth rate of Nile tilapia fed different levels of *Vitex agnus-castus* (VAC) extract.

**Figure:**
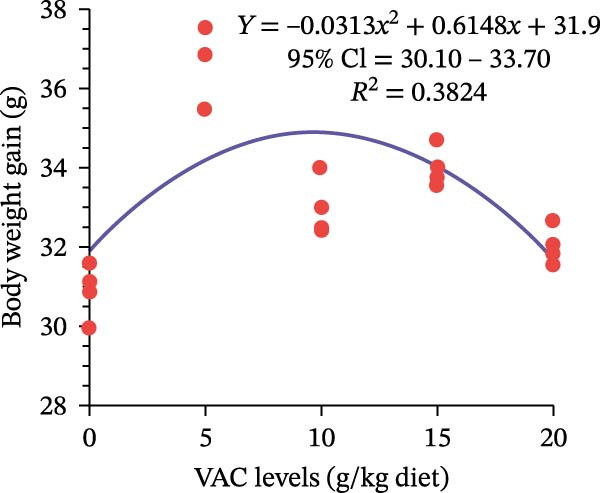
(A)

**Figure:**
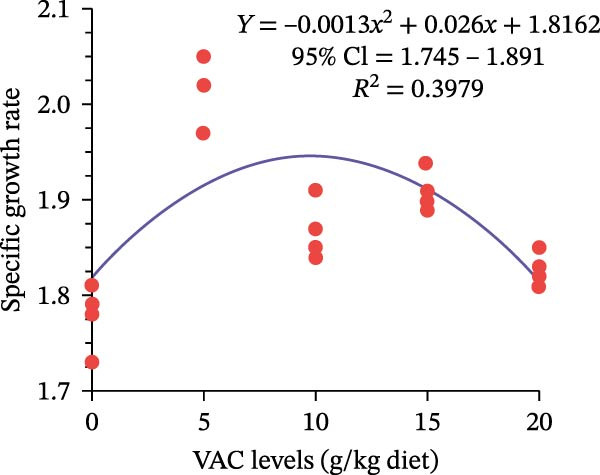
(B)

**Table 2:** Effect of increasing levels of *Vitex agnus-castus* extract on growth performance and feed utlization of Nile tilapia, *Oreochromis niloticus*.

Parameter	*Vitex agnus-castus* level (g kg^–1^ diet)					*p* Value
	0	5	10	15	20	
Final weight (g)	30.89 ± 0.34^c^	36.86 ± 0.49^a^	32.97 ± 0.36^b^	33.99 ± 0.25^b^	32.03 ± 0.23^b^	<0.0001
Weight gain (g)	22.29 ± 0.34^c^	28.26 ± 0.49^a^	24.37 ± 0.36^b^	25.40 ± 0.25^b^	23.43 ± 0.23^bc^	<0.0001
Average daily gain (g day^–1^fish^–1^)	0.310 ± 0.00^c^	0.393 ± 0.01^a^	0.339 ± 0.01^b^	0.353 ± 0.00^b^	0.325 ± 0.00^bc^	<0.0001
Body weight index (%)	259.2 ± 3.98^c^	328.7 ± 5.68^a^	283.4 ± 4.22^b^	295.4 ± 2.96^b^	272.5 ± 2.71^bc^	<0.0001
Specific growth rate (% day^–1^)	1.78 ± 0.02^c^	2.02 ± 0.02^a^	1.87 ± 0.02^b^	1.91 ± 0.01^b^	1.83 ± 0.01^b^	<0.0001
Feed intake (g)	50.23 ± 1.50	53.34 ± 1.46	50.62 ± 0.62	53.96 ± 1.38	48.76 ± 0.96	0.0448
Feed conversion ratio	2.25 ± 0.05^a^	1.89 ± 0.05^b^	2.08 ± 0.02^ab^	2.06 ± 0.04^ab^	2.08 ± 0.06^ab^	0.0006
Protein efficiency ratio (%)	1.48 ± 0.03^c^	1.77 ± 0.04^a^	1.61 ± 0.02^b^	1.57 ± 0.04^b^	1.61 ± 0.04^b^	0.0008

*Note: N* = 4; means in the same row that are labeled with different letters exhibit significant differences (*p* ≤ 0.05).

### 3.2. Digestive Enzymes

The lipase and amylase activity of *O. niloticus* given varying concentrations of VAC was demonstrated by the data in Figure 2A,B. Of all the groups, *O. niloticus* in the VAC_5_ group had the highest (*p* ≤ 0.05) activity in the two digestive enzymes of amylase and lipase.

Figure 2Effect of increasing levels of *Vitex agnus-castus* (VAC) extract on (A) lipase and (B) amylase activities of Nile tilapia, *Oreochromis niloticus*.

**Figure:**
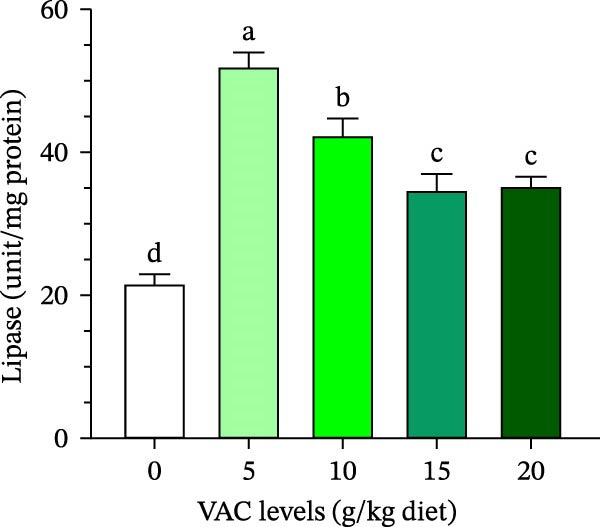
(A)

**Figure:**
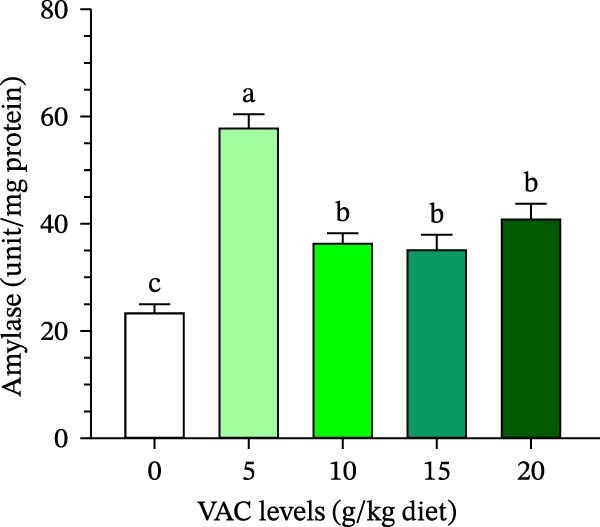
(B)

### 3.3. Hematological Parameters

Table 3 examines the effect of different dietary levels of VAC extract (0, 5, 10, 15, and 20 g/kg diet) on hematological parameters in Nile tilapia. The Hb, RBCs, PCV, MCV, PLT, and WBCs of *O. niloticus* significantly increased in the VAC_10_ and VAC_20_ groups among all groups (*p* ≤ 0.05; Table 3). However, the highest values for Hb, RBCs, PCV, and WBCs occurred at 10 g/kg, suggesting enhanced blood health. The MCH and MCHC values did not differ significantly among all treatments (*p* > 0.05). However, BP as comprehensive physiological parameter did not show any significant changes.

**Table 3:** Effect of increasing levels of *Vitex agnus-castus* extract on hematological parameters of Nile tilapia, *Oreochromis niloticus*.

Parameter	*Vitex agnus-castus* level (g kg^–1^ diet)					*p* Value
	0	5	10	15	20	
Hemoglobin (g dL^–1^)	6.87 ± 0.03^c^	7.27 ± 0.44^b^	8.17 ± 0.44^a^	7.17 ± 0.61^b^	8.13 ± 0.18^a^	0.0125
Red blood cells (×10^6^ µL^–1^)	1.05 ± 0.01^d^	1.24 ± 0.01^c^	1.44 ± 0.00^a^	1.34 ± 0.02^b^	1.21 ± 0.03^c^	<0.0001
Packed cell volume (%)	17.07 ± 0.49^c^	20.90 ± 0.35^b^	24.63 ± 1.19^a^	21.97 ± 1.13^b^	23.03 ± 0.94^a^	0.0014
Mean corpuscular volume (µ^3^)	152.3 ± 2.02^b^	162.5 ± 3.32^ab^	167.2 ± 0.06^a^	161.9 ± 0.78^ab^	157.3 ± 1.10^b^	0.0020
Mean corpuscular hemoglobin (pg)	57.03 ± 1.45	56.80 ± 1.04	55.90 ± 1.56	57.30 ± 1.21	53.70 ± 0.29	0.2758
Mean corpuscular hemoglobin concentration (%)	34.40 ± 1.15	35.87 ± 0.87	33.10 ± 0.56	32.90 ± 1.21	34.57 ± 0.90	0.2559
Platelets (×10^3^ µL^–1^)	37.17 ± 0.17^c^	43.00 ± 2.31^b^	54.00 ± 1.00^a^	49.00 ± 3.46^ab^	51.57 ± 0.64^ab^	0.0007
White blood cells (×10^3^ µL^–1^)	69.27 ± 0.26^d^	87.87 ± 0.49^c^	108.2 ± 1.08^a^	81.67 ± 1.99^c^	100.5 ± 0.37^b^	<0.0001
Blood performance (BP)	4.58 ± 0.20	4.80 ± 0.39	4.98 ± 0.08	4.75 ± 0.35	4.91 ± 0.44	<0.07

*Note: N* = 8; means in the same row that are labeled with different letters exhibit significant differences (*p* ≤ 0.05).

### 3.4. Serum Biochemical Parameters

Serum transaminases (ALT and AST), GLU, TG, and creatinine were significantly decreased in *O. niloticus* fed different levels of VAC extract, especially at levels of 5 g (VAC_5_) and 10 g (VAC_10_) kg^–1^ diet. Meanwhile, serum TP and ALB were significantly increased in *O. niloticus* in VAC_5_, VAC_10_, and VAC_15_ groups among all groups (*p* ≤ 0.05; Table 4). However, serum TCH was significantly increased in VAC extract at level‐dependent manner compared to the control group. Serum GLB and UA were not significantly affected (*p* ≥ 0.05; Table 4).

**Table 4:** Effect of increasing levels of *Vitex agnus-castus* extract on serum biochemical parameters of Nile tilapia, *Oreochromis niloticus*.

Parameter	*Vitex agnus-castus* level (g kg^–1^ diet)					*p* Value
	0	5	10	15	20	
Alanine aminotransferase (u L^–1^)	72.33 ± 1.45^a^	59.00 ± 0.58^d^	61.83 ± 0.44^c^	66.50 ± 2.02^b^	69.50 ± 0.87^ab^	<0.0001
Aspartate transaminase (u L^–1^)	31.00 ± 1.15^a^	19.00 ± 0.58^c^	23.33 ± 1.45^b^	23.67 ± 1.45^b^	30.33 ± 1.45^a^	0.0002
Total protein (g dL^–1^)	2.85 ± 0.03^b^	3.73 ± 0.19^a^	3.63 ± 0.24^a^	3.55 ± 0.09^a^	3.23 ± 0.03^ab^	0.0082
Albumin (g dL^–1^)	1.38 ± 0.17^b^	2.37 ± 0.22^a^	2.33 ± 0.34^a^	2.45 ± 0.03^a^	1.97 ± 0.03^ab^	0.0173
Globulin (g dL^–1^)	1.47 ± 0.17	1.37 ± 0.07	1.30 ± 0.10	1.10 ± 0.06	1.27 ± 0.03	0.1762
Glucose (g dL^–1^)	134.3 ± 4.06^a^	91.50 ± 0.87^b^	96.50 ± 3.18^b^	130.0 ± 6.93^a^	129.0 ± 2.31^a^	<0.0001
Total cholesterol (g dL^–1^)	75.33 ± 5.21^b^	119.7 ± 5.67^a^	135.0 ± 8.08^a^	134.7 ± 3.53^a^	137.3 ± 2.19^a^	<0.0001
Triglycerides (g dL^–1^)	278.3 ± 2.41^a^	122.3 ± 6.36^b^	166.5 ± 4.91^b^	248.0 ± 5.40^a^	260.0 ± 1.36^a^	<0.0001
Creatinine (g dL^–1^)	0.470 ± 0.02^a^	0.320 ± 0.03^b^	0.347 ± 0.04^b^	0.420 ± 0.05^ab^	0.467 ± 0.03^a^	0.0104
Uric acid (g dL^–1^)	1.70 ± 0.12	1.53 ± 0.09	1.50 ± 0.10	1.75 ± 0.03	1.70 ± 0.12	0.3066

*Note: N* = 8; means in the same row that are labeled with different letters exhibit significant differences (*p* ≤ 0.05).

### 3.5. Histomorphometry Characteristics of the Intestine

The intestinal histomorphometry results of *O. niloticus* fed varying concentrations of VAC extract are displayed in Table 5. When *O. niloticus* was fed varying amounts of VAC extract, their muscle, submucosa thickness, villi length, and intestinal breadth all expanded considerably (*p* < 0.05), particularly in the VAC_5_ and VAC_10_ groups compared to other groups. As compared to the control group, the intensity of villi was significantly (*p* < 0.05) decreased in fish fed VAC_5_ than in other VAC‐treated groups.

**Table 5:** Effect of increasing levels of *Vitex agnus-castus* extract on intestinal histometric properties of Nile tilapia, *Oreochromis niloticus*.

Parameter	*Vitex agnus-castus* level (g kg^–1^ diet)					*p* Value
	0	5	10	15	20	
Muscular thickness (μm)	3.33 ± 0.12^b^	5.07 ± 0.22^a^	4.88 ± 0.26^a^	4.56 ± 0.18^a^	5.14 ± 0.30^a^	<0.0001
Submucosa thickness (μm)	1.65 ± 0.09^b^	3.33 ± 0.22^a^	2.76 ± 0.21^a^	1.86 ± 0.14^b^	2.00 ± 0.12^b^	<0.0001
Villi measurements						
Length (μm)	16.60 ± 0.71^c^	24.92 ± 0.68^a^	22.13 ± 0.82^b^	15.59 ± 0.32^c^	18.59 ± 0.64^bc^	<0.0001
Width (μm)	7.96 ± 0.27^c^	10.54 ± 0.42^a^	9.54 ± 0.21^a^	8.13 ± 0.32^b^	9.45 ± 0.24^a^	<0.0001
Intensity (villi mm^−2^)	10.80 ± 0.22^a^	7.13 ± 0.22^c^	9.07 ± 0.36^b^	9.07 ± 0.41^b^	9.33 ± 0.23^b^	<0.0001

*Note: N* = 6; means in the same row that are labeled with different letters exhibit significant differences (*p* ≤ 0.05).

### 3.6. Muscular Chemical Composition

In the present study, *O. niloticus* fed a 5 g VAC extract kg^–1^ (VAC_5_) showed a significant increase of dry matter and crude protein. However, the fat content of fish muscle was significantly increased in those fed a 10 g VAC kg^–1^ among all treatments (*p* ≤ 0.05; Table 6). The energy content (EC) of fish muscles and ash levels did not differ significantly across treatments (*P* ≥ 0.05; Table 6).

**Table 6:** Effect of increasing levels of *Vitex agnus-castus* extract on muscular proximate chemical composition (dry weight basis) of Nile tilapia, *Oreochromis niloticus*.

Parameter	*Vitex agnus-castus* level (g kg^–1^ diet)					*p* Value
	0	5	10	15	20	
Dry matter (%)	13.41 ± 0.18^c^	16.38 ± 0.10^a^	14.90 ± 0.07^b^	14.80 ± 0.15^b^	14.61 ± 0.28^b^	<0.0001
Protein (%)	90.76 ± 0.08^b^	91.12 ± 0.31^a^	89.63 ± 0.01^c^	90.95 ± 0.23^b^	89.96 ± 0.45^c^	0.0102
Fat (%)	3.76 ± 0.17^ab^	3.46 ± 0.21^b^	4.76 ± 0.04^a^	3.73 ± 0.09^ab^	4.50 ± 0.47^a^	0.0166
Ash (%)	5.49 ± 0.24	5.42 ± 0.12	5.61 ± 0.03	5.32 ± 0.17	5.53 ± 0.09	0.6963
Energy content (MJ 100 g^–1^ DM)	2294 ± 8.30	2291 ± 2.09	2307 ± 1.30	2298 ± 3.38	2305 ± 8.52	0.2728

*Note: N* = 6; means in the same row that are labeled with different letters exhibit significant differences (*p* ≤ 0.05).

### 3.7. Flesh Quality Parameters

All measured flesh quality indices in the current study, such as WHC, SL, DL, and FLR at different times (0 h, 1 h, and 2 h), were significantly lower for *O. niloticus* fed different levels of VAC extract than the control group. This decrease was dependent on VAC levels (*p* < 0.05; Table 7). Among all groups, fish fed 5 and 10 g VAC kg^–1^ had the highest flesh quality parameters.

**Table 7:** Effect of increasing levels of *Vitex agnus-castus* extract on flesh quality parameters of Nile tilapia, *Oreochromis niloticus*.

*Vitex agnus-castus* Level (g kg^–1^ diet)	Water holding capacity (%)	Stored loss (%)	Drip loss (%)	Frozen leakage rate (%)		
				0 h	1 h	2 h
0	7.35 ± 0.18^a^	2.53 ± 0.14^b^	7.83 ± 0.52^a^	1.37 ± 0.13^a^	3.29 ± 0.17^a^	7.44 ± 0.17^a^
5	5.91 ± 0.59^b^	3.86 ± 0.35^a^	7.62 ± 0.07^a^	1.01 ± 0.03^b^	2.24 ± 0.09^b^	5.13 ± 0.34^b^
10	5.02 ± 0.24^bc^	2.93 ± 0.25^b^	6.00 ± 0.12^b^	1.32 ± 0.14^a^	2.54 ± 0.07^b^	5.72 ± 0.34^b^
15	4.73 ± 0.28^c^	2.50 ± 0.18^b^	6.77 ± 0.37^b^	0.99 ± 0.03^b^	2.37 ± 0.08^b^	5.15 ± 0.25^b^
20	4.30 ± 0.39^c^	2.24 ± 0.07^b^	6.88 ± 0.30^b^	0.97 ± 0.09^b^	1.86 ± 0.07^c^	5.61 ± 0.53^b^
*p* Value	0.0001	0.0412	0.0232	0.0110	0.0001	0.0142

*Note: N* = 6; means in the same column that are labeled with different letters exhibit significant differences (*p* ≤ 0.05).

## 4. Discussion

Due to their special qualities, which include appetite stimulation, growth promotion, immune response enhancement, aphrodisiacs, anti‐stress, and anti‐pathogenic properties, the use of aromatic plants as feed additives is strongly advised in the aquaculture industry [57, 17]. In the current study, *O. niloticus* fed 5 g VAC kg^–1^ diet showed a substantial enhancement in growth performance and feed efficiency metrics. In the same line, Gholampour et al. [33] [32] observed that the addition of VAC extract at the level of 5 g kg^−1^ diet of zebrafish, *D. rerio*, generated the lowest FCR and the highest SGR compared to the control group, or even increasing the supplementation level to 15 g VAC kg^−1^ diet [33]. Additionally, Rashmeei et al. [34] found that the goldfish fed a diet supplemented with 1.5% VAC extract had the lowest FCR and the maximum body WG. *C. carpio* fed different levels of VAC extract experienced an enhancement of growth performance and feed utilization [36]. This improvement could be the result of the numerous active chemical compounds found in VAC extract, which have anti‐inflammatory, antioxidant, antimicrobial, and energy metabolism modulator as indicated in Table 1. Moreover, it could enhance appetite, increase the activity of digesting enzymes, and improve intestinal microorganisms balance [58, 31]. Moreover, VAC fruit extract supplementation could upregulate the expression of genes linked to appetite and growth, such as growth hormone (GH), insulin‐like growth factor (IGF‐1), and ghrelin genes [34].

The positive effects of VAC extract on the growth and feed utilization of *O. niloticus* are similar to those obtained by other herbal extracts on Nile tilapia, such as elephant’s foot, *Elephantopus scaber* extract [59, 60], *Mitracarpus scaber* leaves extract [24], *Annona squamosa* leaves extract [22], *Moringa oleifera* aqueous extract [17], guava, and *Psidium guajava* leaves extract [25]. Medicinal plants have the ability to increase appetite and WG by increasing the release and activities of digestive enzymes [61]. In accordance, Nile tilapia fed artichoke leaf extract‐supplemented diet expressed higher growth performance and lower feed intake, and the best results were obtained at 2% supplementation level [62].

The current findings showed that *O. niloticus* fed a diet supplemented with 5 g VAC kg^–1^ diet significantly increased the tested digestive enzymes (lipase and amylase) among all treatments, as shown in Figure 2. The same findings were also reported on *O. niloticus* fed 6 g of *Tridax procumbens* leaves extract kg^–1^ diet [20], fed 20 g of *Aegle marmelos* fruit extract kg^–1^ [63], and those fed caper (*Capparis spinosa*) extracts [57]. Khanzadeh et al. [64] found that feeding *O. niloticus* with 1% and 2% *Laurencia caspica* extract substantially boosted digestive enzyme activities compared to the control group. In addition, Bello et al. [65] hypothesized that higher digestive activity boosted the absorption of vitamins, cofactors, and enzymes, resulting in improved growth performance. Plant‐based bioactive substances that stimulate immunity have the potential to improve gut microbiota and elevate digestive enzymes in both rainbow trout (*Oncorhynchus mykiss*) and African catfish (*Clarias gariepinus*) when their diets include extracts of clove and basil leaves, respectively [66, 67].

Hematological and blood biochemical markers are generally used to assess fish’s general health and nutritional status [44]. The current findings revealed significant improvements of hematological and biochemical parameters, especially at levels of 5 and 10 g VAC. The beneficial effects of VAC are directly due to its bioactive compounds [31], including diterpenoids, flavonoids, triterpenes, tannins, iridoids, and alkaloids, which stimulated hematopoietic organs in fish. Similarly to the current findings, Rashmeei et al. [34] found that goldfish given 1.5% VAC extract had the highest RBCs, Hb, and PCV among all groups. In the same line, beneficial effects of different medicinal plant extracts on haemato‐biochemical properties were previously reported in *O. niloticus*, such as *M. scaber* leaves [20], *M. oleifera* [17], baobab, and *Adansonia digitate* [21]. In this study, fish fed with herbal extracts showed enhanced hematological parameters, likely promoting erythropoiesis, increasing oxygen delivery, fortifying stress resistance, and ultimately improving growth performance [13].

Liver is the main metabolic organ in the fish body, when it develops damage, the level of functional enzymes (ALT, AST, and lactate dehydrogenase [LDH]) rises in blood [18]. In the current findings, *O. niloticus* given varying amounts of VAC displayed that serum ALT and AST were dramatically reduced. In the same vein, Rashmeei et al. [34] reported that raising the supplementation levels of VAC extract reduced the serum ALT, AST, and LDH levels of goldfish, with the lowest values occurring in those that received 1.5% VAC extract. Khoris et al. [36] reported a significant reduction of serum ALT, AST, TP, and ALB in *C. carpio* fed different levels of VAC extract and challenged with *Vibrio anguillarum*. These findings reflected the protective role of VAC on the hepatic tissue, which could relate to the presence of natural antioxidants and other bioactive substances in VAC extract [31]. In addition, *O. niloticus* fed varying levels of VAC in the current investigation showed considerably higher serum TP and ALB than those fed the VAC‐free diet. These findings are directly related to better liver status in *O. niloticus* fed varying levels of VAC in the present study.

In the present study, serum TCH was significantly increased; however, serum TG and creatinine significantly decreased in *O. niloticus* fed the low levels of VAC extract. In keeping with the present findings, Khoris et al. [36] found that the liver and kidney function enzymes of *C. carpio* against *V. anguillarum* challenge were highly improved by VAC extract. Through its antioxidant properties, in vivo injection of VAC for 5 days protects against organs damage mediated by lipopolysaccharide [68, 69]. GLU, an important stress indicator, is continuously evaluated via in vivo blood analysis following acute or chronic exposure to various stressors [70]. In many animals, increasing the production and uptake of GLU by tissues provides the energy needed to cope with stress [71]. *O. niloticus* was given several concentrations of VAC extract in the current investigation, resulting in a considerable drop in blood GLU concentration. Goldfish‐fed VAC showed the similar results [34]. This is most likely caused by a decrease in cortisol‐induced gluconeogenesis and a conversion of glycogen to GLU in tissues [72].

Intestinal health is an important practice for maintaining better feed utilization [73]. Thus, there is considerable interest in developing new feed formulations and feeding practices to promote the development and health of fish gastrointestinal tract [61]. Goblet cells count and intestinal villi length have a direct effect on fish digestion and nutritional absorption [74], as well as absorption area capacity; therefore, they are considered good measures of intestinal health [75]. Furthermore, an increase in the villi’s length may improve the small intestine’s surface area for nutritional interaction and improve nutrition absorption and growth of fish [76]. Thus, histomorphological analysis is essential in aquaculture studies to evaluate the effects of feed ingredients on hepatic and intestinal health [77]. In the present study, *O. niloticus* fed 5–10 g VAC kg^–1^ showed significant improvement of histomorphometric parameters of the intestine among all treatments. The same findings were observed in the intestine of *O. niloticus* fed different levels of baobab, *A*.*digitata* fruit extract, without any inflammatory signs [21]. These results have been previously validated in the intestine of *O. niloticus* treated with different medicinal plant extracts [78, 79] and similarly observed in species like largemouth bass, *Micropterus salmoides* [80], *Pangasianodon hypophthalmus* [81], and spotted sea bass, *Lateolabrax maculatus* [82]. Additionally, the helpful effects on fish intestinal histology could be linked to the antioxidant and anti‐inflammatory properties of phytonutrients present in medicinal plants [83, 84].

In respect to the muscle composition, *O. niloticus* given a 5 g VAC extract kg^–1^ demonstrated a substantial increase in dry matter and crude protein and a decrease in fish muscle fat content. *O. niloticus* fed varying concentrations of *E. scaber* extract [60] and European sea bass, *Dicentrarchus labrax*, fed 100 g kg^–1^ red grape polyphenol extract [85], both showed similar effects on the chemical composition of their dorsal muscles. This could be attributed to the beneficial effects of biologically active components in VAC extract, which improve nutrition absorption in the digestive system [33, 69, 86]. According to Du et al. [87], dietary supplementation with flavonoids efficiently controls the metabolism, transport, and absorption of lipids. Additionally, it raises MAPK8 and NF‐κβ c‐Rel levels and promotes IGF‐2 expression, which is reflected in the growth and enhancement of protein synthesis in the animal. These biologically active substances can induce higher organismal metabolism, which improves lipid mobilization and breakdown, as well as protein synthesis efficiency [88]. As a result, the fish will be healthier and have good flesh quality, offering nutritious and cheaper aquatic products for human consumption [89]. Recently, Mehrim et al. [90] also stated the same effects of caraway essential oil (CEO) on the chemical composition of dorsal muscles of *O. niloticus*. Furthermore, dietary pure anise oil enhanced muscle nutrient content, such as protein level in *O. niloticus* [91].

In the present study, the enhancement of muscle proximate composition was reflected in the flesh quality, whereas fish fed 5 g and 10 g kg^–1^ diet experienced significant improvement of flesh quality parameters. In the same line, Mehrim et al. [90] reported enhancement of flesh quality parameters of *O. niloticus* fed a 0.1 g kg^–1^ diet of CEO. In addition, dietary supplementation of *Silybum marianum* increased muscle fiber growth and GH gene expression [92]. The obtained improvement could be attributed to flavonoids and diterpenes, which improve growth and protein synthesis by increasing nutrient bioavailability [84, 88]. Similarly, dietary polyphenol‐rich extract (100 mg kg^–1^) significantly prevented lipid oxidation and improved flesh quality parameters of European sea bass [85].

## 5. Conclusion

Based on the obtained results, this study is considered a pioneer work on the impact of VAC extract on *O. niloticus*. It showed that supplementation with 5–10 g kg^–1^ diet of VAC extract significantly enhanced the growth performance, digestive enzyme activities, immunological and physiological responses, intestinal histometric, chemical makeup, and quality of the meat of *O. niloticus*. In addition, from the quadratic regression analysis, it could be confirmed that the predicted maximum VAC extract level for *O. niloticus* fingerling growth and feed utilization is 9.81 g kg^–1^ diet. However, deep investigation about the mode of action, especially on the molecular levels, could be conducted to optimize the use of VAC extract in fish diet.

## Author Contributions

**Ahmed Ismail Mehrim**: experimental design, writing, revising the manuscript. **Mohamed Moaaz Refaey**: experimental design, sample collection, data analysis, revising the manuscript. **Abdallah Tageldein Mansour and Ehab El-Haroun:** results interpretation, revising the manuscript, visualization. **Osama Awad Zenhom**: experimental conduction, sample collection. **Hamada Antar Areda:** experimental design, results interpretation, revising the manuscript, culturing fish, collecting samples.

## Funding

This work has no funding support.

## Ethics Statement

The handling and experimental procedures involving all animals were carried out following the “Ethics and Guidelines of Animal Care and Use Committee,” Mansoura University, Egypt (Number: MU‐ACUC: AGR.R.25.11.16).

## Conflicts of Interest

The authors declare no conflicts of interest.

## Data Availability

The data that support the findings of this study are available from the corresponding author upon reasonable request.
